# Mechanisms of Karyotypic Diversification in *Ancistrus* (Siluriformes, Loricariidae): Inferences from Repetitive Sequence Analysis

**DOI:** 10.3390/ijms241814159

**Published:** 2023-09-15

**Authors:** Kevin Santos da Silva, Larissa Glugoski, Marcelo Ricardo Vicari, Augusto César Paes de Souza, Alberto Akama, Julio Cesar Pieczarka, Cleusa Yoshiko Nagamachi

**Affiliations:** 1 Cytogenetics Laboratory, Center for Advanced Biodiversity Studies Science Institute Biological, Federal University of Pará, Belém 66075-110, Brazil; kevin.snts.slv@gmail.com (K.S.d.S.); julio@ufpa.br (J.C.P.); 2 Fish Cytogenetics Laboratory, Federal University of São Carlos, São Carlos 13565-905, Brazil; larissaglugoski23@gmail.com; 3 Laboratory of Chromosome Biology: Structure and Function Department of Structural Biology, Molecular and Genetic, University of Ponta Grossa State, Ponta Grossa 84010-330, Brazil; vicarimr@uepg.br; 4 Laboratory of Amazon Ichthyofauna Study, Federal Institute of Pará, Abaetetuba 68440-000, Brazil; acps1203@gmail.com; 5 Department of Zoology, Paraense Emilio Goeldi Museum, Belém 66040-170, Brazil; aakama@gmail.com

**Keywords:** Ancistrini, cytogenomics, gene families, karyotypic evolution, non-coding RNAs

## Abstract

*Ancistrus* is a highly diverse neotropical fish genus that exhibits extensive chromosomal variability, encompassing karyotypic morphology, diploid chromosome number (2n = 34–54), and the evolution of various types of sex chromosome systems. Robertsonian rearrangements related to unstable chromosomal sites are here described. Here, the karyotypes of two *Ancistrus* species were comparatively analyzed using classical cytogenetic techniques, in addition to isolation, cloning, sequencing, molecular characterization, and fluorescence in situ hybridization of repetitive sequences (i.e., 18S and 5S rDNA; U1, U2, and U5 snDNA; and telomere sequences). The species analyzed here have different karyotypes: *Ancistrus* sp. 1 (2n = 38, XX/XY) and *Ancistrus cirrhosus* (2n = 34, no heteromorphic sex chromosomes). Comparative mapping showed different organizations for the analyzed repetitive sequences: 18S and U1 sequences occurred in a single site in all populations of the analyzed species, while 5S and U2 sequences could occur in single or multiple sites. A sequencing analysis confirmed the identities of the U1, U2, and U5 snDNA sequences. Additionally, a syntenic condition for U2-U5 snDNA was found in *Ancistrus*. In a comparative analysis, the sequences of rDNA and U snDNA showed inter- and intraspecific chromosomal diversification. The occurrence of Robertsonian rearrangements and other dispersal mechanisms of repetitive sequences are discussed.

## 1. Introduction

Loricariidae is a monophyletic group that includes about 1042 species of fish endemic to the neotropical region, distributed from Panama to Argentina [1]. Currently, this family is organized into six subfamilies, with Hypostominae being the most diverse and widely distributed, harboring 500 species belonging to 45 genera [1].

*Ancistrus* Kner, 1854 (Hypostominae, Ancistrini) is one of the most diverse and well-distributed neotropical fish genera, with a similar distribution of the family [1]. Currently, there are seventy-six valid species, along with numerous lineages not formally identified, that are related to *Ancistrus* [1,2,3]. This group presents large karyotypic diversity, mainly with extensive variation in the diploid number (2n = 34–54 chromosomes) and karyotypic morphologies; additionally, the occurrence of different types of sex chromosome systems (e.g., single: XX/XY, XX/X0, and ZZ/ZW; and multiple: XX/XY_1_Y_2_, ZZ/ZW_1_W_2_, and Z_1_Z_1_Z_2_Z_2_/Z_1_Z_2_W_1_W_2_) represents another important aspect of chromosomal evolution in *Ancistrus* [4,5,6,7,8,9,10].

Robertsonian (Rb) rearrangements, particularly Rb fusion, are recognized as the primary mechanism responsible for the high variability in the diploid chromosome number (2n) observed in *Ancistrus*. In Hypostominae, most species have karyotypes with 2n = 52, this 2n being considered an ancestral condition for the Ancistrini tribe [11]. Interestingly, most of the *Ancistrus* species analyzed have karyotypes with 2n < 52, suggesting a tendency towards 2n reduction due to the occurrence of double-strand breaks (DSB) and DNA fusions throughout the karyotypic evolution of this group of fish [9,12,13].

In fish, especially in *Loricariidae* species, several studies have associated the occurrence of chromosomal reorganization events with unstable chromosomal sites that are rich in different groups of repetitive sequences [9,12,14,15,16]. Repetitive sequences represent the majority of eukaryotic genomes and have numerous physiological and structural functions, and can be grouped in tandem repeats (e.g., gene families) or distributed in dispersed groups throughout the genome (e.g., transposable elements) [17,18].

Due to the repetitive nature, the chromosomal sites formed by these groups of DNA sequences are considered unstable, prone to DSB and rearrangements, representing important markers for the analysis of karyotypic evolution [19,20,21]. Gene families (e.g., ribosomal genes: rDNA; and small nuclear DNA: snDNA) are classes of repetitive DNA useful for the study of chromosomal evolution in fish, showing different patterns of organization and distribution in the genomes of related species [22,23,24,25,26,27]. In Loricariidae, a syntenic and single-site organization between the 18S and 5S rDNA genes represents a plesiomorphic condition [11], while the synteny break and occurrence of multiple sites of these genes is frequent in Ancistrini, showing mechanisms of chromosomal differentiation involving this group of sequences [9,13,26]. However, information about the chromosomal organization of U snDNA sequences is limited to a few species of Loricariidae [26,28,29,30].

The U snDNA are genes that transcribe non-coding RNAs involved in the spliceosome arrangement, the structure responsible for the maturation of messenger RNA (mRNA) in eukaryotes [31]. Sequences of these genes are organized in tandem repeats and used in comparative cytogenetic analysis [21,22,25,27,29,32]. Comparative analyses of in situ localization and molecular characterization demonstrate that, unlike other groups of genes encoding non-coding RNAs, U snRNA genes show remarkable similarity with orthologous copies [27]. These genes may present distinct chromosomal organizations in different groups of species, occurring in single, multiple sites or clusters formed by different gene families (e.g., snRNA and rRNA genes) [26,27,28,29,32,33,34,35]. Furthermore, genomic data demonstrate that pseudogenes and non-functional copies of U snDNA occur in fish genomes [27,29], indicating the participation of these repetitive sequences in chromosomal reorganization, including transposition events.

In this study, a comparative molecular characterization of U snDNA sequences, as well as in situ localization of repetitive sequences (i.e., U1 and U2 snDNAs, 18S and 5S rDNAs, and telomeric sequences) were performed to better understand the chromosomal diversification in two species of *Ancistrus* from the Amazon region.

## 2. Results

### 2.1. Karyotypes in Ancistrus

**Figure 1:**
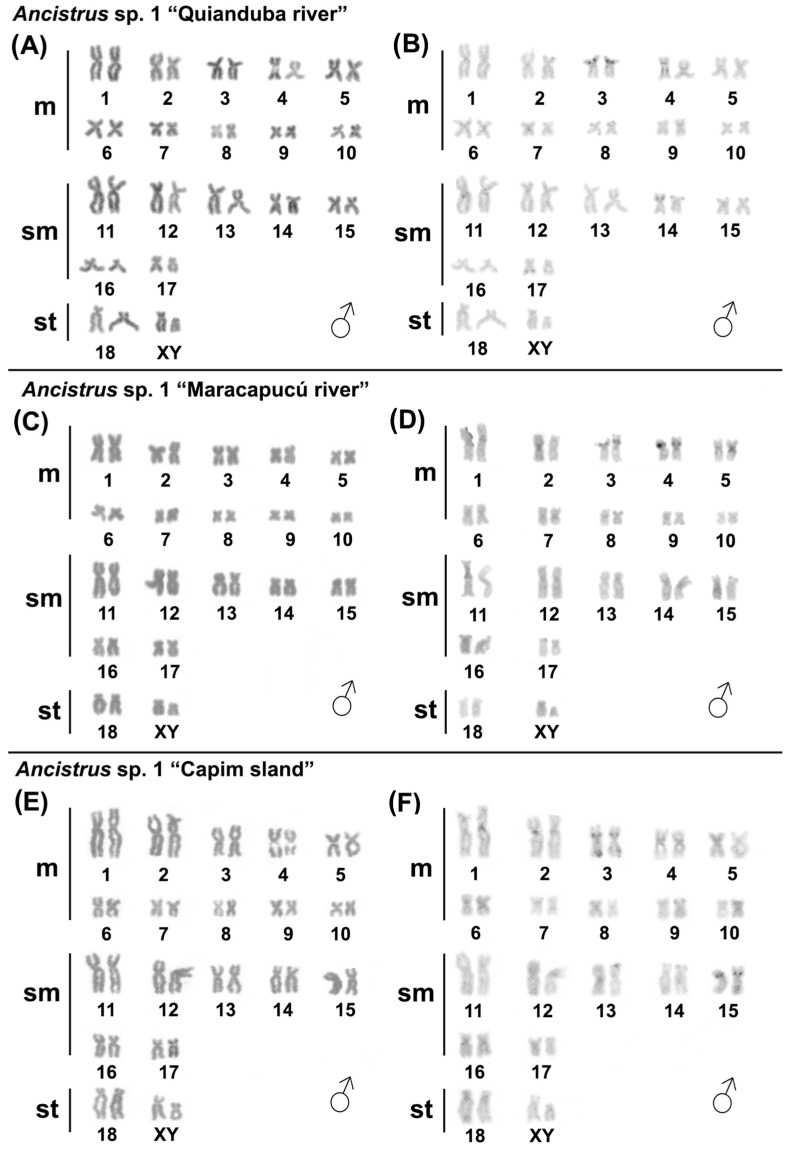
Karyotypes of male individuals of the different populations of *Ancistrus* sp. 1 analyzed in this study: in (**A**,**B**) population of “Quianduba river”, (**C**,**D**) population of “Maracapucú river”, and (**E**,**F**) population of “Capim island” after conventional staining and C-banding, respectively. Scale: 10 µm.

**Figure 2:**
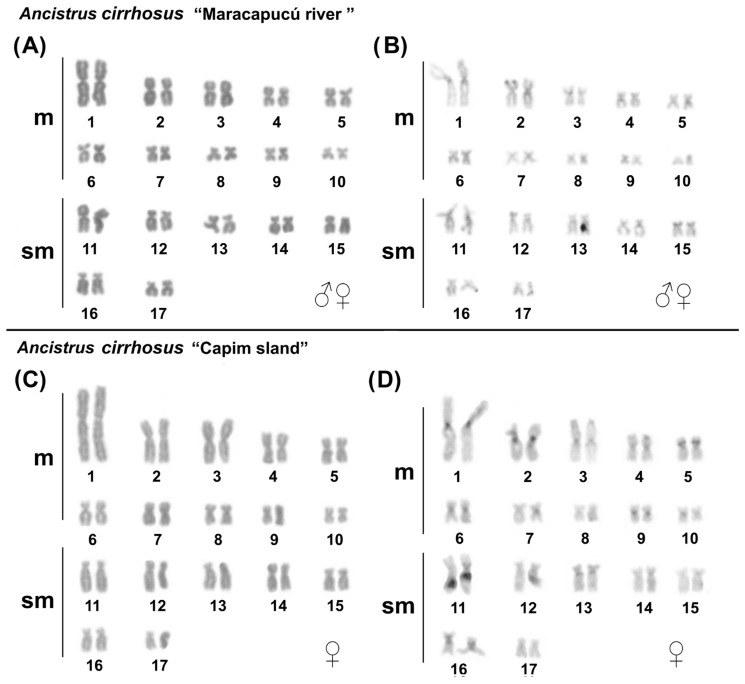
Karyotypes of the different populations of *Ancistrus cirrhosus* analyzed in this study: in (**A**,**B**) population from “Maracapucú river” and (**C**,**D**) population from “Capim island” after conventional staining and C-banding, respectively. (♀) female and (♂) male karyotypes. Scale: 10 µm.

Karyotypes of *Ancistrus* sp. 1 the “Quianduba river” and *Ancistrus cirrhosus* populations from “Maracapucú river” and “capim island” previously described [9] were confirmed in this study (Table 1, Figure 1A,B and Figure 2A–D). Karyotypes of *Ancistrus* sp. 1 populations from “Maracapucú river” and “Capim island” were analyzed for the first time in this study, showing 2n = 38 chromosomes (KF = 20 m + 14 sm + 4 st). Size heteromorphisms between a pair of chromosomes were observed in all male *Ancistrus* sp. 1, which confirms the occurrence of XX/XY sex chromosomes in this species; the X chromosome is medium subtelocentric, and the Y is small subtelocentric. C-banding showed the occurrence of centromeric/pericentromeric constitutive heterochromatin (CH) blocks in only a few pairs of chromosomes. CH regions were not evident on the sex chromosomes in any of the populations of *Ancistrus* sp. 1 analyzed in this study (Figure 1C–F).

### 2.2. In Situ Localization of Repetitive Sequences in Ancistrus

**Figure 3:**
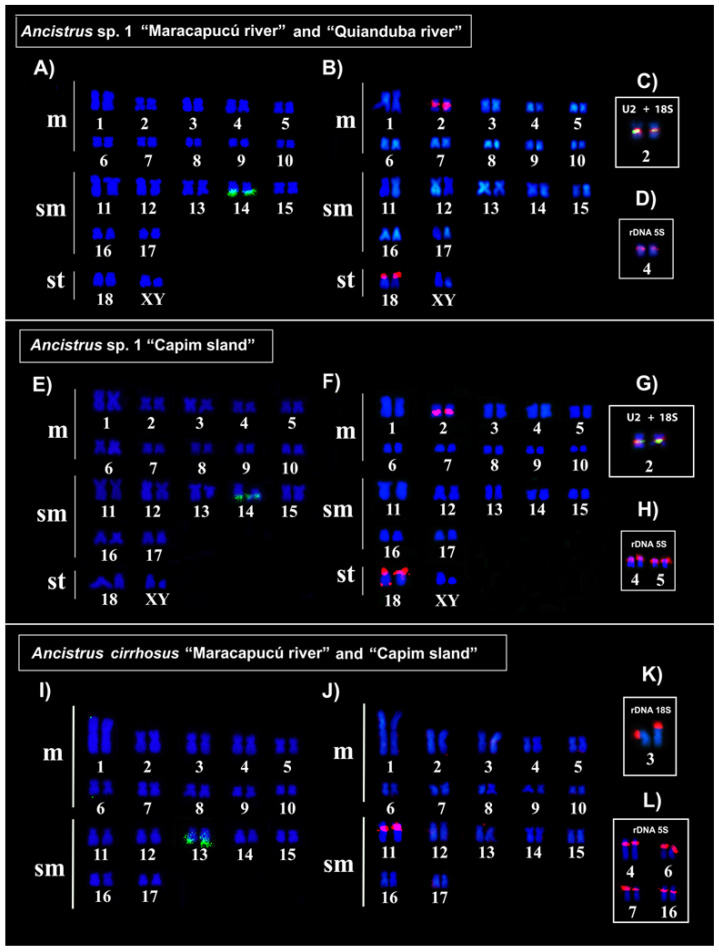
Fluorescence in situ hybridization indicating the physical location of repetitive sequences in the species/populations of *Ancistrus* analyzed in this study. Probes were labeled with FITC (green) and CY3 (red), and chromosomes were counterstained with DAPI (blue). (**A**) U1 snDNA (green); (**B**) U2 snDNA (red); (**C**) double FISH with 18S rDNA (green) and U2 (red), and (**D**) 5S rDNA (red) in *Ancistrus* sp. 1 “Quianduba river” and “Maracapucú river”; (**E**) U1 snDNA (green); (**F**) U2 snDNA (red); (**G**) double FISH with 18S rDNA (green) and U2 (red); and (**H**) 5S rDNA (red) in *Ancistrus* sp. 1 “Capim Island”; (**I**) U1 snDNA; (**J**) U2 snDNA; (**K**) 18S rDNA, and (**L**) 5S rDNA in populations of *Ancistrus cirrhosus* “Maracapucú river” and “Capim Island”. Scale: 10 µm.

The in situ mapping of U1 snDNA (probe 157-bp) and U2 snDNA (probe 190-bp) showed a non-syntenic organization, in addition to different positions and numbers of clusters among the karyotypes of *Ancistrus* species/populations analyzed in this study (Figure 3, Table 1). In all populations of *Ancistrus* sp. 1, the U1 snDNA occurred in the distal region of pair 14q (Figure 3A,E), while U2 snDNA occurred in multiple sites in the proximal region of pair 2 and in the distal region of pair 18p in populations of *Ancistrus* sp. 1 “Quianduba river” and “Maracapucú river” (Figure 3B); additionally, U2 snDNA sites in the distal region of the long arm of only one of the homologues of the 18q pair were uniquely identified in *Ancistrus* sp. 1 “Capim island” (Figure 3F). In *Ancistrus cirrhosus*, the mapping of U1 snDNA showed the presence of this repetitive sequence in the distal region of pair 13q (Figure 3I), while U2 snDNA occurred in the pericentromeric region of pair 11 in all populations analyzed (Figure 3J).

The in situ localization of 18S and 5S rDNA genes in populations of *Ancistrus* sp. 1 “Quianduba river” (Figure 3C,D), *Ancistrus cirrhosus* “Maracapucú river” and “Capim island” (Figure 3K,L) previously analyzed [9] was confirmed in this study. All populations of *Ancistrus* sp. 1 presented 18S rDNA clusters in the proximal region of pair 2 (Figure 3C,G), while 5S rDNA occurred in the interstitial region of pair 4 in the populations of “Maracapucú river” and “Quianduba river” (Figure 3D), and in multiple clusters in the interstitial region of pairs 4 and 5 in the population of “Capim island” (Figure 3H) (Table 2). Interstitial telomere sequences (ITS) were not evident in *Ancistrus* sp. 1 “Maracapucú river” and “Capim island” (Supplementary Figure S1), similarly to what was previously observed for *Ancistrus* sp. 1 “Quianduba river” [9].

### 2.3. U snDNA Sequences from Ancistrus

The set of primers allowed the correct isolation of units of the U1 snDNA (157-bp) and U2 snDNA (190-bp). Additionally, by using the U2 snDNA primers, a U5 snDNA copy (114-bp) was also detected, present between two U2 snDNA units, and separated by non-transcribed spacer (Table 2 and Table 3; Supplementary Figure S2). The analyses using the BLASTn, Rfam, and RepeatMasker tools confirmed the identities of the U snDNA sequences obtained here as corresponding to the U1, U2, and U5 spliceosomal RNA genes and revealed remarkable similarity with other sequences obtained from the genomes of other neotropical fish (Table 2 and Table 3). Besides that, the presence of other repetitive elements associated with the U1, U2, and U5 snDNA sequences obtained from the genome of *Ancistrus* sp. 1 (2n = 38, XX/XY) was not evident.

**Figure 4:**
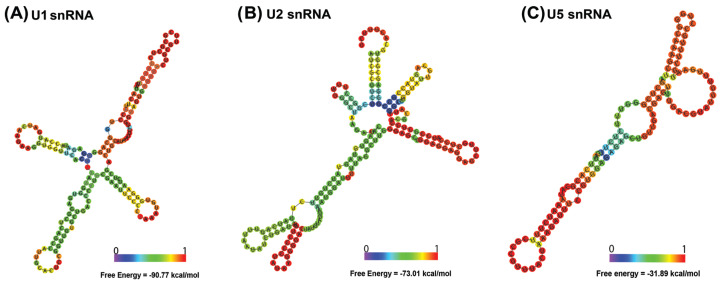
Prediction of secondary structures of U snRNA obtained from the genome of *Ancistrus* sp. 1 (2n = 38, XX/XY): in (**A**) the secondary structure of the U1 snRNA, in (**B**) the U2 snRNA, and in (**C**) the U5 snRNA.

The construction of secondary structures for the U1, U2, and U5 snRNA obtained in this study were similar to those observed for other neotropical fish [27,29]. The U1 snRNA showed a secondary structure with four loops (Figure 4A). The U2 snRNA had a five-loop structure (Figure 4B), while the U5 snRNA had a two-loop structure (Figure 4C).

## 3. Discussion

The subfamily Hypostominae represents one of the most diverse and widely distributed lineages of Loricariidae. In this group, most species have karyotypes with 2n = 52; on the other hand, representatives of the tribe Ancistrini (e.g., genus *Ancistrus*) seem to follow different paths, displaying very different karyotypes in chromosome morphology and diploid numbers when compared to the proposed ancestral karyotype for the family Loricariidae (2n = 54) [11,36]. Some studies suggest that DSB and chromosomal rearrangements are recurrent in the karyotypes of these lineages, leading to their extensive chromosomal diversity [9,12,13,16].

The karyotypic diversity in the genus *Ancistrus* includes a reduction in 2n (2n = 34–54) and evolution of different types of the heteromorphic sex chromosome systems (for a review, see [9,10]). This diversity has been explained by the occurrence of Rb rearrangements (Rb), mainly fusions, related to chromosomal regions rich in repetitive sequences [9,12,13,16]. In this study, the analyzed species with lower 2n when compared to the ancestral karyotype proposed for Loricariidae (2n = 54) and Ancistrini tribe (2n = 52) probably results from successive fusion events. The occurrence of ITS is suggestive of fusions [12]; however, in the analysis carried out previously [9] and in the present study, ITS was not found in any karyotype of the analyzed samples. Alternatively, the occurrence of these rearrangements has been corroborated to the detriment of the absence of ITS due to the accumulation of other groups of repetitive sequences in unstable sites prone to DBS, which may explain the occurrence of these rearrangements in these karyotypes [9,13,26,28,37]. Furthermore, the absence of ITS in rearranged karyotypes is commonly related to the loss of these sequences during fission/fusion events or the degeneration of these interstitial sites throughout the evolution of karyotypes [38].

18S and 5S rDNA genes are part of gene families extensively used in the comparative cytogenetic analysis in fish, showing the occurrence of chromosomal differentiation [9,14,24,26,28,37,38]. In Loricariidae, a syntenic organization is considered a plesiomorphic condition [11,39]. In *Ancistrus*, the synteny break between these gene families and the multiple 5S rDNA sites occurrences are common features (for a review, see [9,13]). Our data agree with this observation, showing intra- and interspecific variations in the position and number of 18S and 5S rDNA sites among the analyzed species, suggesting the involvement of this group of repetitive sequences in inter- and intraspecific karyotypic dispersion and diversification mechanisms in *Ancistrus* [9,12].

In a comparative analysis, 5S rDNA is notably more dynamic than 18S rDNA in the genomes of *Ancistrus* [9,12,13]. Both of these groups of rDNA are associated with the occurrence of DSB and chromosomal rearrangements in different organisms, including fish [12], mammals [40], and plants [41]. The occurrence of multiple 5S rDNA clusters in the pericentromeric region of the *Ancistrus* sp. 1 (2n = 38, XX/XY) and *Ancistrus cirrhosus* (2n = 34) suggests of the involvement of this gene family in chromosome fusions observed in the genus [9,12,13]. For example, Barros et al. [12] proposed an Rb fusion event to justify the diploid number reduction observed in *Ancistrus* sp. (2n = 52) with multiple ITS-associated 5S rDNA clusters present in the centromeric region in a metacentric pair. Recently, multiple 5S rDNA sites associated with different groups of microsatellites located in the pericentromeric region were also proposed as unstable chromosomal sites and related to Rb fusions to explain the 2n reduction observed in the karyotype of *Ancistrus* sp. 2 (identified here as *Ancistrus cirrhosus*) [9]. These analyses suggest a close relationship between rDNA sites and chromosomal rearrangements in *Ancistrus*, as observed in other loricariids [9,12,13,16,26,28]. These data support the hypothesis that rDNA sequences represent regions of evolutionary breakpoints (EBR) due to the reuse of these sites in chromosomal rearrangements in Loricariidae and other eukaryotes [9,12,16,19,20,40].

Interestingly, comparative mapping of 5S rDNA showed intraspecific variation in *Ancistrus* sp. 1 (2n = 38, XX/XY), demonstrating multiple sites in the population from the “Capim island” and single site in the populations from the “Quianduba river” and “Maracapucú river” (Figure 4D,H). Dispersion involving transposable elements or non-homologous recombination events are important mechanisms related to the emergence of additional 5S rDNA sites in fish [23]. The 5S rDNA clusters are formed by tandem repetitive units with coding regions (~120 bp) separated by non-transcribed spaces (NTS) of varying sizes due to the accumulation of other repetitive elements [23,42]. The recurrent association of these genes with mobile elements is described in several fish species and may play a key role in the dispersion of this sequence by facilitating the occurrence of DSB, transpositions, and non-homologous recombination [23]. Therefore, in addition to involvement in chromosomal rearrangements, we consider that the participation of 5S rDNA in transposition events and non-homologous recombination may represent plausible mechanisms to explain the dispersion of these sequences to new chromosomal sites in different populations of *Ancistrus* sp. 1 (2n = 38, XX/XY) and *Ancistrus cirrhosus* (2n = 34), similarly to what has already been described in other fish groups [9,12,13,14,15,26,28].

The U snRNA genes represent another group of tandem repeat sequences used in comparative chromosomal analyses in fish [25,27,29]. Unlike other genes that express non-coding RNAs, U *snRNA* gene families exhibit remarkable similarity in base sequences when copies obtained from different groups of organisms are compared [25,27,29]. The conservation of these sequences suggests high selective pressure against mutations and alterations in the final structure of RNA transcribed from these genes in eukaryotes. This observation is valid for the U1, U2, and U5 snDNA sequences obtained from the genome of *Ancistrus* sp. 1 (2n = 38, XX/XY) that are significant similarity in base sequences and secondary structures when compared with orthologous copies obtained from the genomes of other neotropical fish (Table 2 and Table 3; Figure 4) [29].

At the chromosomal level, U snDNA sequences tend to occur in individualized sites [25,26,27,28,29,33]. Additionally, variations in the number of sites and multiple dispersed clusters in the genome have also been observed [29,43]. For example, Schott et al. [29] described for the first time the sequences and in situ localization of snDNA sequences U1, U2, U4, U5, and U6 in *Ancistrus* and demonstrated the presence of these sequences (except U4 and U6 snDNAs) in single sites in *Ancistrus* sp. (2n = 50), *A. aguaboensis* (2n = 50), and A. cf. *multispinis* (2n = 52), suggesting stability and non-involvement of these sequences in chromosomal rearrangements in these species. Here, in situ mapping of U1 and U2 snDNA demonstrated a non-syntenic condition for these sequences, similarly to that described for other species of *Ancistrus* [29]; on the other hand, multiple U2 snDNA clusters were observed for the first time in *Ancistrus* sp. 1 (2n = 38, XX/XY), suggesting their dispersion through the genomes.

The emergence of multiple U snDNA clusters has been documented in other fish groups and has been related to retrotranspositions [29,32]. However, the U1 and U2 snDNA sequencing data here obtained from the genome of *Ancistrus* sp. 1 (2n = 38, XX/XY) did not show any association of these genes with mobile elements. These data agree with what was observed for sequences of these genes obtained from other species of *Ancistrus* [29]. Alternatively, the presence of pseudogenes and defective copies (e.g., without promoter sequences) suggest the occurrence of transpositions involving U snDNA genes in other fish species, as observed for U1, U2, and U6 in *Apareiodon* sp. [27] and U4 snDNA in *Ancistrus* [29]. These data indicate that transposition may represent plausible mechanism to justify the dispersion of these sequences in the genomes of these organisms.

Additionally, our in situ localization data demonstrates colocalization between U2 snDNA and 18S rDNA sequences in the proximal region of pair 2 in all populations of *Ancistrus* sp. 1 (2n = 38, XX/XY) (Figure 3C,G). Comparative in situ localization studies have shown the occurrence of chromosomal sites formed by more than one multigenic family in different groups of fish [29,33,34,35], in the present study. The 45S rDNA sites are formed by the 18S, 5.8S, and 28S rDNA genes separated by NTS of varying sizes due to the accumulation of different repetitive sequences commonly associated with heterochromatin. Heterochromatic regions are formed by different groups of repetitive sequences, which could be related to the occurrence of DSB, transpositions, and non-allelic recombinations, allowing variations in the location and number of sites of these sequences. These characteristics suggest instability for these chromosomal regions, which may explain the dispersion of U2 snDNA sequences to new sites in *Ancistrus* sp. 1 (2n = 38, XX/XY), as proposed for other groups of organisms [22,32,40].

## 4. Materials and Methods

### 4.1. Samples

**Figure 5:**
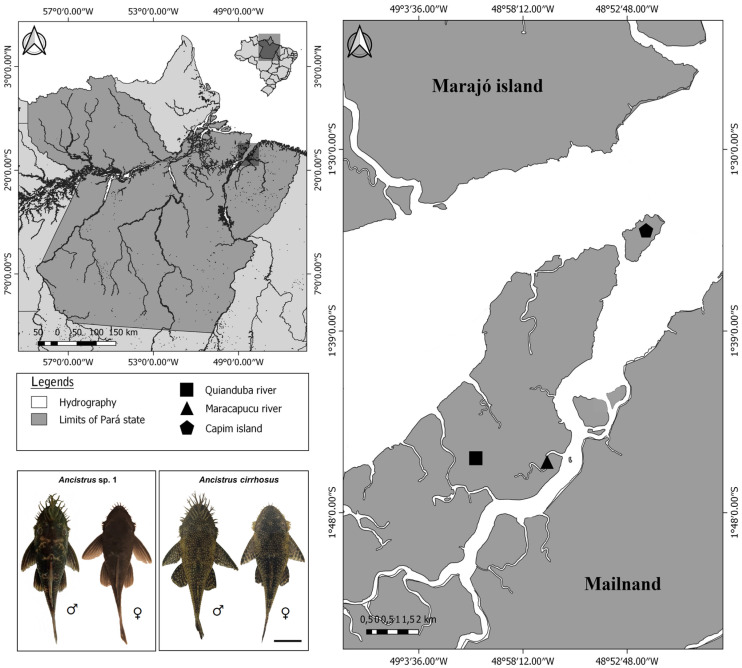
Geographic location of *Ancistrus* sp. 1 and *Ancistrus cirrhosus* sample collection points analyzed in this study. This map was built using Q-GIS software version 3.4.5 using the Instituto Brasileiro de Geografia e Estatística (IBGE) data. Photos of the specimens by Kevin Santos da Silva.

Samples belonging to two species identified as *Ancistrus* sp. 1 and *Ancistrus cirrhosus* (Valenciennes, 1836) collected at the Tocantins-Araguaia River basin, Brazil, were analyzed (Figure 5). Details on the number of individuals, collection points, location, sex, and deposit in a zoological collection are described in Table 4. The Chico Mendes Institute for Biodiversity Conservation authorized the collections (permanent license number 13,248). The Cytogenetics Laboratory of the Federal University of Pará has licenses for transport (number 19/2003) and use of animals in scientific research (52/2003) granted by the Ministry of the Environment, Brazil. The Animal Ethics Committee of the Federal University of Pará, Brazil, approved this research (permission 68/2015). The specimens analyzed in this study are deposited in the Ichthyology collection of the Center for Advanced Biodiversity Studies, Belém, Brazil.

### 4.2. Isolation and Amplification of Repetitive Sequences

Genomic DNA was obtained from tissue (muscle) samples using the PureLink Genomic DNA Kit (Promega, Madison, WI, USA) following the manufacturer’s instructions. Analyzed sequences were isolated via Polymerase Chain Reaction (PCR) using primers to 18S and 5S rDNA sequences and U1 and U2 snDNA, described by Gross et al. [44], Martins and Galetti Jr. [45], and Azambuja et al. [27], respectively. PCR mixes consisted of 1× Buffer Solution (200 mM Tris pH 8.4, 500 mM KCl), MgCl_2_ (50 mM), dNTPs (10 mM), primers (10 mM), 1U Taq DNA Polymerase (5 U/μL), and genomic DNA (100 ng/μL). The amplification conditions consisted of initial denaturation at 95 °C for 5 min, 35 cycles of 95 °C for 1 min, 60 °C for 45 s, and 72 °C for 1 min, and final extension at 72 °C for 5 min. All PCR products were checked on electrophoresis using 1% agarose gel.

### 4.3. Cloning, Sequencing, and Characterization of Repetitive Sequences

PCR products amplified using primers for the U1 and U2 snDNAs were purified on Wizard^®^ SV Gel and PCR Clean-up System (Promega Madison, WI, USA) and cloned using pGEM^®^-T Easy Vector Systems (Promega, Madison, WI, USA). The base sequences were obtained in an ABI Prism 3500 Genetic Analyzer sequencer (Applied Biosystems, Waltham, MA, USA), edited, and analyzed in the Geneious 7.1.3 software [46]. The identities of these sequences were confirmed using the Basic Local Alignment Search Tool (BLASTn) from the National Center for Biotechnology Information (http://blast.ncbi.nlm.nih.gov/Blast.cgi, accessed on 10 February 2022), Rfam (https://rfam.xfam.org, accessed on 15 March 2023) and Repeat Masker (www.repeatmasker.org, accessed on 15 March 2023).

The secondary structures of the complete U2 and U5 snDNA sequences were generated using the RNAfold tool available in the ViennaRNA 2.0 package [47]. For U1 snDNA (incomplete sequence), the secondary structure was constructed from alignment with functional copies of U1 snDNA from *Apareiodon* sp. (GenBank accession number: MZ645211.1), *Colossoma macropomum* (GenBank accession number: XR_005008802.1) and *Pygocentrus nattererii* (GenBank accession number: XR_005130783.1) using the RNAalifold tool [40] available via the ViennaRNA 2.0 package [48].

### 4.4. Obtaining and Analyzing Metaphases

Mitotic metaphases were obtained through direct extraction from kidney cells, after treatment with colchicine solution (0.025%) [49]. The animals were previously anesthetized with eugenol and then sacrificed to obtain cells. Chromosomes were analyzed via conventional staining (Giemsa diluted in phosphate buffer pH 6.8), C-banding [50], and fluorescence in situ hybridization (FISH) using sequences of 18S and 5S rDNA, U1 and U2 snDNAs, and telomeric probes [51].

### 4.5. Probes and Fluorescent In Situ Hybridization (FISH)

The 18S rDNA, U1 snDNA (clone with 157-bp), and U2 (clone with 190-bp) sequences (Table 3 and Table 4) were obtained via PCR from genomic DNA of *Ancistrus* sp. 1 (2n = 38, XX/XY), while the 5S rDNA sequences were obtained from the *Ancistrus aguaboensis* genome (GenBank accession number MT018470; [13]) and used as probes in FISH experiments. Probes were labeled with biotin or digoxigenin by nick translation (Invitrogen^®^) or via PCR using incorporation of digoxigenin-11-dUTP (Roche Applied Science^®^). Telomere probes were obtained via PCR with the incorporation of Biotin-11-dUTP (Invitrogen^®^) without the use of template DNA [52]. FISH was performed according to Pinkel et al. [51], with modifications, under the following stringency conditions: 2.5 ng/μL of probe, 50% formamide, 2xSSC, 10% Dextran Sulfate, and hybridization at 42 °C for 16 h. Hybridization signals were detected using Streptavidin Alexa Fluor 488 (Molecular Probes, Carlsbad, CA, USA) and anti-digoxigenin rhodamine Fab fragments (Roche Applied Science, Penzberg, Germany). Chromosomes were counterstained with 4′6-diamidino-2-phenylindole (DAPI, 0.2 μg/mL) in Vectashield mounting medium (Vector, Burlingame, CA, USA).

### 4.6. Images and Karyotypic Analysis

About 30 metaphases of each sample were analyzed to determine the diploid number (2n), chromosome arm number (FN), karyotypic formula (KF), chromosome banding, and FISH experiments. Giemsa-stained images were obtained using an Olympus BX41 microscope coupled to a CCD 1300QDS digital camera (Spectral Imaging) and analyzed using GenASIs software version 7.2.7.34276 (AppliedSpectral Imaging, Carlsbad, CA, USA) from ASI version 7.2.7.34276 (Applied Spectral Imaging). FISH images were obtained using a Nikon H550S microscope and analyzed using Nis-Elements software version 4.0 (Melville, NY, USA). All images were adjusted using Adobe Photoshop CS6 software. The karyotypes were organized following the chromosomal morphology classification proposed by Levan et al. [53] in metacentric (m), submetacentric (sm), and subtelocentric (st). When counting the FN, m, sm, and st chromosomes were considered as having two arms.

## 5. Conclusions

In this study, our results demonstrate extensive diversity of location and number of sites for the analyzed repetitive sequences, mainly 5S rDNA and U2 snDNA, in species of the genus *Ancistrus* from the Amazon region. A comparative analysis of the karyotypes suggests the occurrence of Robertsonian rearrangements, transpositions, and non-homologous recombination events as mechanisms related to karyotypic diversification among the *Ancistrus* species analyzed.

## Figures and Tables

**Table 1:** Comparative description of chromosomal diversity among *Ancistrus* species/populations analyzed in this study.

Species	Populations	2n	FN	KF	SC	rDNA		snDNA		Ref.
						18S	5S	U1	U2	
*Ancistrus* sp. 1	Quianduba ^(^*^)^	38	72	20 m + 14 sm + 4 st	XX/XY	2q	4p	14q	2, 18p	1, 2
	Maracapucú									
	Capim island						4p, 5p		2, 18p, homologue 18q	
*Ancistrus cirrhosus*	Maracapucú ^(^*^)^	34	68	20 m + 14 sm	Not found	3p	4p, 6p, 7p, 16	13q	11	1, 2
	Capim island ^(^*^)^									

Legend: 2n: diploid number; FN: Fundamental Number; KF: karyotypic formula; SC: sex chromosomes; rDNA: *ribosomal* genes; snDNA: *small nuclear* RNA genes; (*): samples previously analyzed by Santos da Silva et al. [9]. References: 1: Santos da Silva et al. [9]; 2: Present study.

**Table 2:** Molecular characterization of U snDNA sequences obtained from the genome of *Ancistrus* sp. 1 (2n = 38, XX/XY) using the BLASTn tool in the NCBI database.

Sequences	BLASTn (GenBank)				
	Gene Family	Organism	Ident (%)	E-Value	Access Number
U1 (157-pb) *	*U1* spliceosomal RNA	*Pygocentrus nattereri*	88.61	9-10^−44^	XR_005129869.1
U2 (1112-pb)	*U2* spliceosomal RNA	*Megaleporinus obtusidens*	90.05	2-10^−61^	MT_563075.1
	*U5* spliceosomal RNA	*Astyanax paranae*	92.31	3-10^−31^	MG_963291.1
	*U2* spliceosomal RNA	*Anguilla anguilla*	91.39	2-10^−49^	XR_004763517.1
U2 (190-pb) *	*U2* spliceosomal RNA	*Tachysurus fulvidraco*	87.33	4-10^−38^	XR_007138734.1

Legend: (*): sequences used as probes in FISH experiments.

**Table 3:** Molecular characterization of U snDNA sequences obtained from the genome of *Ancistrus* sp. 1 (2n = 38, XX/XY) using local alignment tool in Rfam database.

Sequences	Rfam					
	Gene Family	Start	End	Bit Score	E-Value	Access Number
U1 (157-pb) *	*U1* spliceosomal RNA	1	196	107.2	6.2-10^−29^	RF00003
U2 (1112-pb)	*U2* spliceosomal RNA	1	191	179.7	7-10^−31^	RF00004
	*U5* spliceosomal RNA	562	676	80.6	2-10^−13^	RF00020
	*U2* spliceosomal RNA	954	1112	142.4	8.1-10^−40^	RF00004
U2 (190-pb) *	*U2* spliceosomal RNA	1	190	119	4.9-10^−26^	RF00004

Legend: (*): sequences used as probes in FISH experiments.

**Table 4:** Details on sampling, sex, number of individuals, and collection sites of specimens of the genus *Ancistrus* analyzed in this study.

Species	Populations	Sex		River	Locality	Voucher ^(#)^	Coordinates
*Ancistrus* sp. 1	Quianduba ^(^*^)^	5♂	2♀	A	Abaetetuba/PA	P4229	S01°45′18.2″/W49°00′38.8″
	Maracapucú	2♂	-♀	B	Abaetetuba/PA	P4264	S01°45′29.2″/W48°56′57″
	Capim island	1♂	-♀	C	Abaetetuba/PA	P4250	S01°34′02.8″/W48°51′49.1″
*Ancistrus cirrhosus*	Maracapucú ^(^*^)^	8♂	1♀	B	Abaetetuba/PA	P4263	S01°45′29.2″/W48°56′57″
	Capim island ^(^*^)^	-♂	1♀	C	Abaetetuba/PA	P4251	S01°34′02.8″/W48°51′49.1″

Legend: A: Quianduba river; B: Maracapucú river; C: Capim island; (-): No samples; (*): samples previously analyzed by Santos da Silva et al. [9]; (♀) female individuals, (♂) male individuals. (#): Ichthyology Collection for Center for Advanced Biodiversity Studies, Belém, Brazil.

## Data Availability

Not applicable.

## Supplementary Materials

The following supporting information can be downloaded at: https://www.mdpi.com/article/10.3390/ijms241814159/s1.
