# Supplementary material for: Mechanisms of Karyotypic Diversification in Ancistrus (Siluriformes, Loricariidae): Inferences from Repetitive Sequence Analysis
Source: Int J Mol Sci. 2023 Sep 15;24(18):14159. doi: 10.3390/ijms241814159 (PMC10532334; doi:10.3390/ijms241814159)
Supplement: Supplementary file 1 [file ijms-24-14159-s001.zip › ijms-2535689-supplementary.pdf]

Title: **Mechanisms of Karyotypic Diversification in Ancistrus (Siluriformes, Loricariidae): Inferences from Repetitive Sequence Analysis**

Authors: Kevin Santos da Silva, Larissa Glugoski, Marcelo Ricardo Vicari, Augusto Paes de Souza, Alberto Akama, Julio Pieczarka and Cleusa Nagamachi.

Supplementary data file:

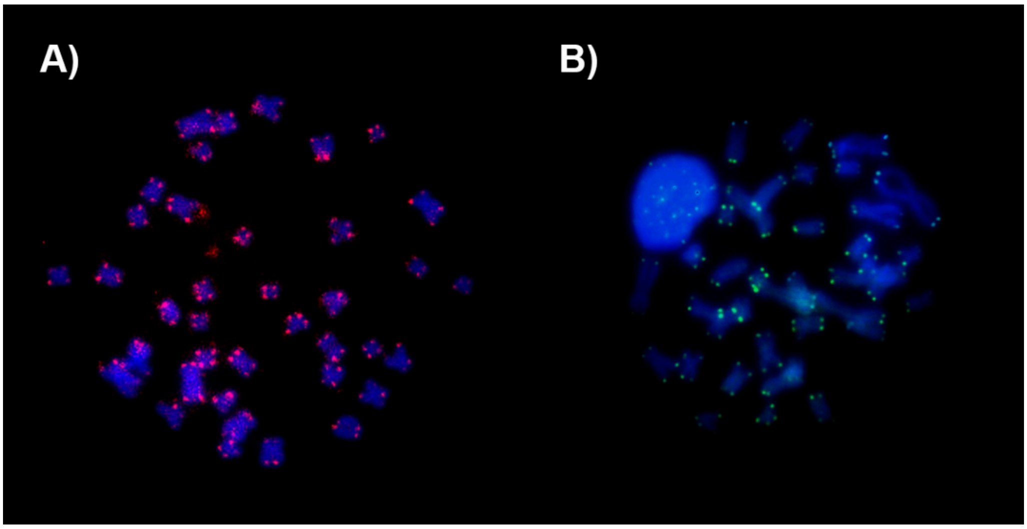

Figure S1: Fluorescent in situ hybridization indicating the physical location of telomere sequences in populations of *Ancistrus* sp. 1 described for the first time in this study. In A) telomeric sequences (red) *Ancistrus* sp. 1 "Maracapucú river", in B) telomeric sequences (green) in *Anicistrus* sp. 1 "Capim Island". Scale: 10 μm.

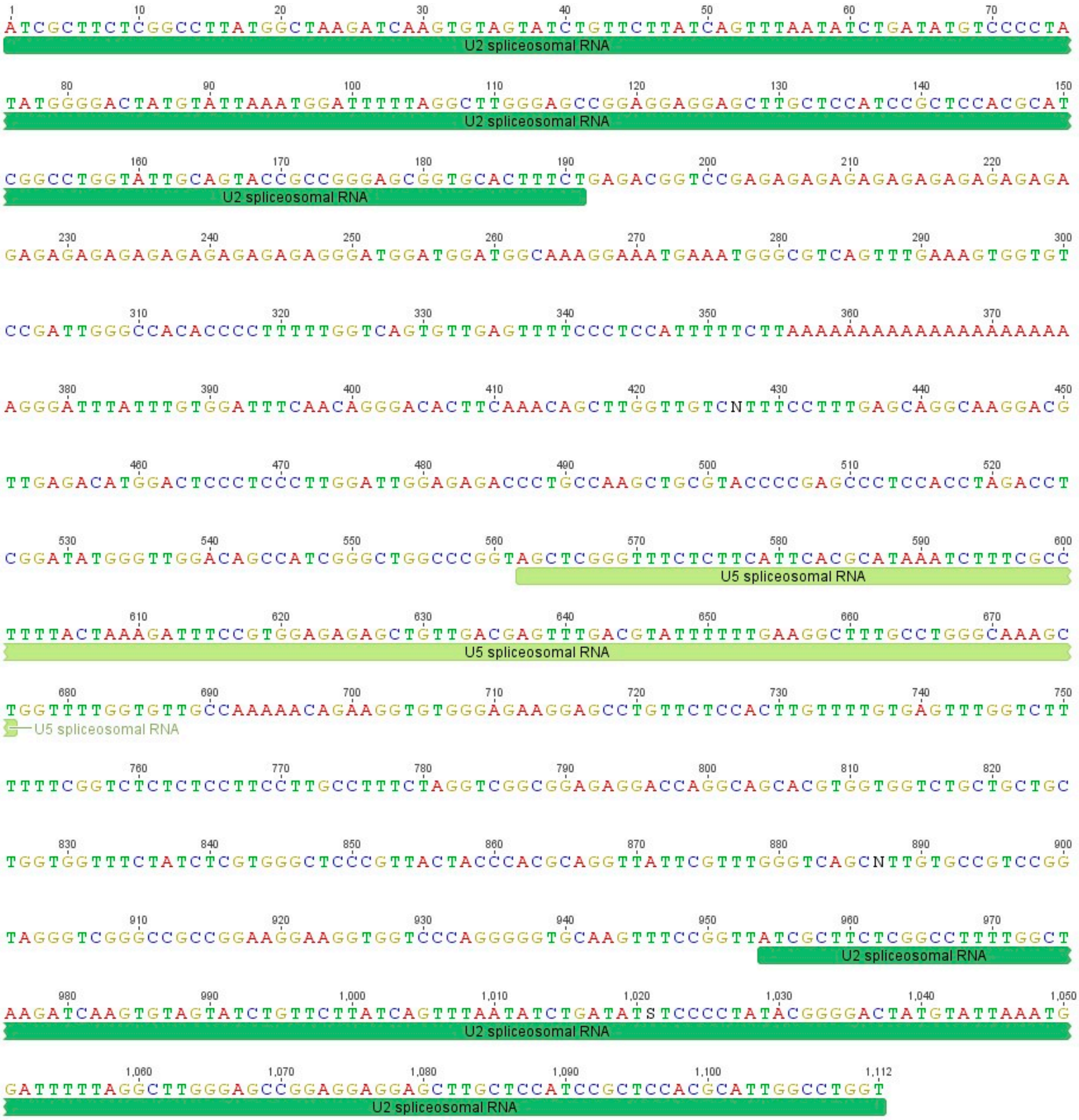

Figure S2: Annotation of complete sequences of U2 and U5 snDNA occurring in synteny in the genome of *Ancistrus* sp. 1 (2n = 38, XX/XY). In dark green annotation of U2 snDNA sequences; in light green the annotation of U5 snDNA sequence.
